# Rice OsANN9 Enhances Drought Tolerance through Modulating ROS Scavenging Systems

**DOI:** 10.3390/ijms242417495

**Published:** 2023-12-15

**Authors:** Yangyang Jia, Xiangyang Gu, Jiaxin Chai, Xiaohong Yao, Shoutao Cheng, Lirui Liu, Saiya He, Yizhuo Peng, Qian Zhang, Zhengge Zhu

**Affiliations:** 1 Ministry of Education Key Laboratory of Molecular and Cellular Biology, Hebei Research Center of the Basic Discipline of Cell Biology, College of Life Sciences, Hebei Normal University, Shijiazhuang 050024, China; jiayangyang9277@163.com (Y.J.); 19832104996@163.com (X.G.); chaijiaxin99@163.com (J.C.); yaoxh1991@163.com (X.Y.); chengshoutao@163.com (S.C.); liulirui202311@163.com (L.L.); a1835637662@163.com (S.H.); pengby3194@foxmail.com (Y.P.); 2 Hebei Collaboration Innovation Center for Cell Signaling and Environmental Adaptation, Hebei Key Laboratory of Molecular and Cellular Biology, Shijiazhuang 050024, China

**Keywords:** rice, OsANN9, drought stress, ROS

## Abstract

Drought is a critical abiotic stress which leads to crop yield and a decrease in quality. Annexins belong to a multi-gene family of calcium- and lipid-binding proteins and play diverse roles in plant growth and development. Herein, we report a rice annexin protein, OsANN9, which in addition to regular annexin repeats and type-II Ca^2+^ binding sites, also consists of a C2H2-type zinc-finger domain. We found that the expression of *OsANN9* was upregulated by polyethylene glycol (PEG) or water-deficient treatment. Moreover, plants that overexpressed *OsANN9* had increased survival rates under drought stress, while both *OsANN9*-RNAi and *osann9* mutants showed sensitivity to drought. In addition, the overexpression of *OsANN9* increased superoxide dismutase (SOD), peroxidase (POD) and catalase (CAT) activities, which regulate reactive oxygen species homeostasis. Collectively, these findings indicate that OsANN9 may function as a positive regulator in response to drought stress by modulating antioxidant accumulation. Interestingly, the setting rates of *osann9* mutant rice plants significantly decreased in comparison to wild-type plants, suggesting that OsANN9 might be involved in other molecular mechanisms in the rice seed development stage.

## 1. Introduction

Drought is one of the major abiotic stresses that affect plant growth and development and therefore crop yield and quality [1]. Rice, a staple food, is needed around the world, and rice production is of great significance to global food security [2]. However, due to climate change and erratic rainfall patterns, drought stress increased globally and led to crop yields decreased [3]. To adapt to drought stress, plants have evolved a series of multifaceted mechanisms which consist of modifying photosynthetic pathways, regulating leaf transpiration [4], upregulating the expression of enzyme genes involved in scavenging reactive oxygen species (ROS) [5], sensing plant hormone signals and so on [6,7]. Thus, identifying drought resistance genes and revealing their molecular mechanisms are crucial goals for crop breeding scientists. 

Annexins belong to a subfamily of Ca^2+^-dependent phospholipid-binding proteins that are prevalent in plants and animals [8,9], and they are highly conserved in evolution. In general, the C-terminal core of annexin contains four annexin repeats, and each repeat has five short α-helices [9,10]. Previous studies have shown that annexins participate in regulating diverse aspects of plant growth and development as well as responses to stress [11,12]. For example, the overexpression of *TdANN6* enhanced salt/osmotic stress tolerance by increasing catalase (CAT), superoxide dismutase (SOD) and peroxidase (POD) activities in durum wheat [13]. *AtANN1* and *AtANN2* affect the growth of primary roots by regulating silique sucrose transport [14]. The expression of *ZmANN33* and *ZmANN35* was up-regulated during seed germination but partially suppressed by chilling, and the heterologous expression of *ZmANN33* or *ZmANN35* in *Arabidopsis* was also beneficial to plasma membrane (PM) recovery under chilling stress [15]. In other examples, AtANN1, a Ca^2+^-permeable transporter, was involved in responses to drought, salinity, abscisic acid (ABA), ROS and cold stress by mediating the accumulation of cytosolic-free Ca^2+^ [16,17,18,19]. AtANN5 accumulated at sites of membrane damage to maintain membrane integrity in pollen grains under osmotic or ionic imbalances [20]. Furthermore, the overexpression of *AtANN8* compromised *RPW8.1*-mediated resistance to powdery mildew and cell death [21]. These results demonstrate that plant annexins are related to diverse physiological pathways.

In recent years, annexins have been reported to be involved in regulating abiotic and biotic stress responses in rice [22]. For example, OsANN5 is reported to be involved in cold stress tolerance at the rice seedling stage [23]. *OsANN1* positively regulates heat stress tolerance by modulating ROS homeostasis [24]. Under normal conditions, OsANN1 interacts with OsHAN1 and promotes JA-Ile to exhibit an inactive form. Upon pathogen infection, OsANN1 and HAN1 are separated, leading to HAN1 degradation and JA-Ile accumulation, thus promoting disease resistance [25]. The overexpression of *OsANN3* significantly increases drought tolerance by regulating ABA-dependent stress response pathways [26]. RNA silencing of *OsANN4* caused hypersensitivity to ABA by modulating ROS production and Ca^2+^ influx [27]. *OsANN10* negatively regulates osmotic stress tolerance by increasing POD and CAT activities to remove excess ROS and by maintaining plasma membrane integrity [28]. However, the physiological roles of most rice annexins are still unclear.

ROS, including singlet oxygen (^1^O_2_), superoxide anions (O_2_^−^), hydrogen peroxide (H_2_O_2_) and hydroxyl radicals (·OH) [29], play an essential role in plant responses to abiotic stress. Under normal conditions, the production and scavenging of ROS remain balanced. However, under drought-stress conditions, this balance is disturbed, resulting in an increase in the production of ROS [30,31], which can damage macromolecules such as proteins, lipids and nucleic acids, ultimately leading to programmed cell death (PCD) [32,33]. Plants have evolved an enzymatic antioxidant defense system to maintain ROS levels. The major ROS scavenging enzymes include SOD, CAT, peroxiredoxin (PrxR), ascorbate peroxidase (APX) and glutathione peroxidase (GPX) [31]. *OsRbohB* encodes an NADPH oxidase and regulates ROS production to enhance drought tolerance in rice [34]. The overexpression of *OsPYL6* can improve drought tolerance by increasing ABA content and reducing H_2_O_2_ accumulation [35]. *OsAAI1* positively regulates drought tolerance by enhancing the activity of ROS scavenging enzymes such as CAT, APX, GPX and GR (glutathione peroxidase) in rice [36]. Interestingly, some reports also suggest that plant annexins respond to drought stress by regulating ROS homeostasis. For instance, the overexpression of *TdANN12* in tobacco improves drought tolerance through the removal of excess ROS [37]. The heterologous expression of *VvANN1* enhances drought tolerance in *Arabidopsis* by increasing the ROS scavenging activities of SOD, POD and CAT [38]. Therefore, plant annexins have critical roles in drought stress and ROS homeostasis, but how annexins are directly involved in this process remains unclear.

In this study, the putative rice annexin *OsANN9* was characterized, and its functions in response to drought stress were explored. We constructed a series of transgenic rice plants including *OsANN9*-OE, *OsANN9*-Ri and *osann9* mutant lines for the experiments used in this study. Our results indicate that *OsANN9* is induced by drought stress, and *OsANN9* participates in the response to drought stress by modulating the level of malondialdehyde (MDA) and the activities of antioxidant enzymes (SOD, POD and CAT). Overall, our results provide a physiological basis for the role of plant annexins during environmental stress.

## 2. Results

### 2.1. OsANN9 Encodes an Annexin Family Member

**Figure 1:**
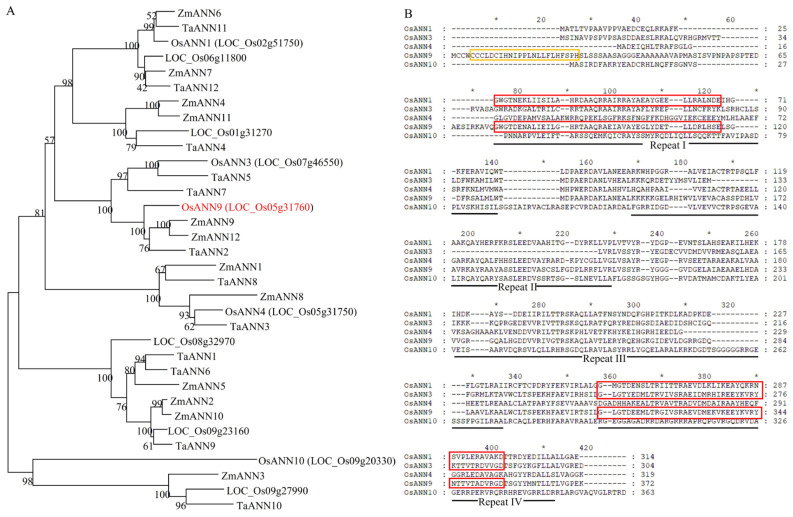
Characterization of OsANN9. (**A**) Phylogenic tree of annexins of rice, maize and wheat. The phylogenetic tree was constructed with MEGA7. All polypeptide sequences of annexins were derived from NCBI. (**B**) Sequence alignment of OsANN9 and other reported annexins in rice. The yellow box indicates the C2H2 domain. The red boxes indicate type-II Ca^2+^-binding sites.

We previously reported that some annexin genes of rice consisting of Os02g51750 (*OsANN1*), Os07g46550 (*OsANN3*), Os05g31750 (*OsANN4*) and Os09g20330 (*OsANN10*) were involved in responses to heat, drought, ABA and osmotic stress [24,26,27,28]. In this study, we have focused on another rice annexin gene, Os05g31760. Based on the amino acid sequence alignment with annexins from other species, it is 73.2% similar to the ZmANN9 sequence of maize, and was named OsANN9 (Figure 1A).

Bioinformatics indicates that there are 10 putative annexin genes in the rice genome, all annexin proteins have a conserved annexin repeat domain and some of them possess type-II Ca^2+^-binding sites; OsANN9 is not an exception. This suggests that OsANN9 might have similar conservative functions as other reported rice annexin proteins [24]. Moreover, OsANN9 also has a distinctive character, such as a zinc-finger-type (C2H2) domain (Figure 1B). This implies that OsANN9 may have some properties that differ from other rice annexin proteins.

**Figure 2:**
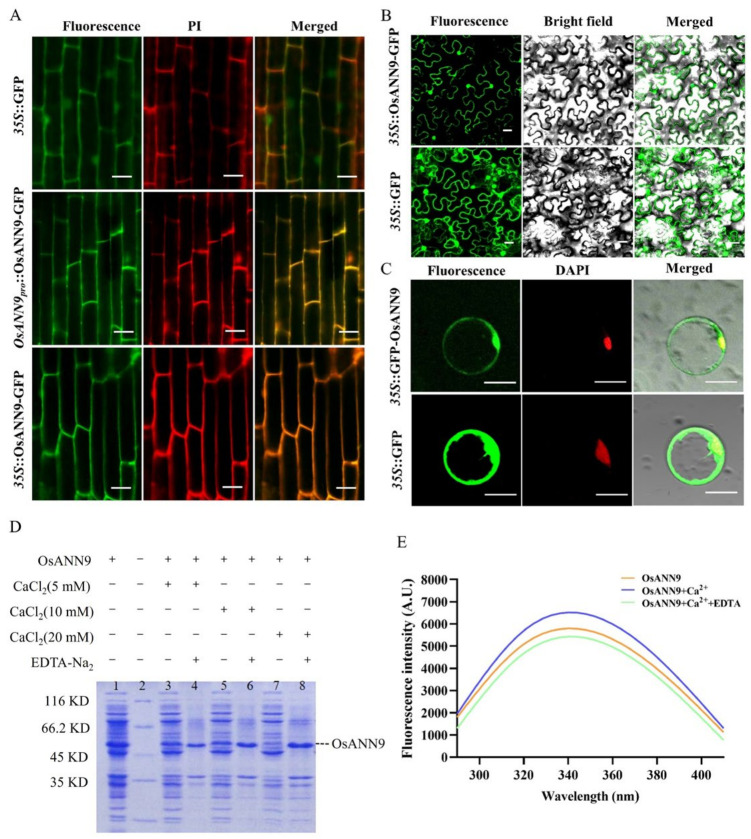
Subcellular localization and Ca^2+^ binding activity of OsANN9. (**A**–**C**). Subcellular localization of OsANN9-GFP in root tips of 3-day-old rice seedlings (**A**), tobacco leaf epidermal cells (**B**), and rice protoplasts (**C**). GFP alone was used as a control. Bar = 20 μm. (**D**) Total protein of the E. coli cultures transformed with pET30a-OsANN9 (lane 1). When different concentrations of CaCl_2_ (5 mM, 10 mM, 20 mM) were added to the supernatants separately, the OsANN9-His protein bound to Ca^2+^ and formed pellets (lanes 3, 5, 7). After adding equal amounts of EDTA-Na_2_ to the pellets, OsANN9-His was released (lanes 4, 6, 8). Lane 2 shows the molecular weight marker. (**E**) Fluorescence intensity of OsANN9 without (blue curve) or with (green curve) 2 mM CaCl_2_. The brown curve shows the fluorescence intensity of OsANN9 with CaCl_2_ and an equal amount of EDTA-Na_2_.

To assay the subcellular localization of OsANN9, the cassettes *35S::OsANN9-GFP*, *OsANN9pro::OsANN9-GFP* and *35S::GFP* were constructed. The corresponding plasmids were introduced into *Agrobacterium tumefaciens* individually and then transformed into rice calli to obtain transgenic rice plants. After that, 3-day-old rice roots of T3 plants were used to determine the subcellular localization of OsANN9-GFP. The results showed that OsANN9-GFP was mainly localized in the plasma membrane and cell periphery in the rice root tip cells, whereas GFP fluorescence alone was ubiquitously distributed in the rice root tip cells (Figure 2A). To further test the transient expression of OsANN9, *35S::OsANN9-GFP* or *35S::GFP* was transformed into rice protoplast or tobacco leaf epidermal cells separately. Interestingly, in rice protoplast and tobacco epidermal cells, the signals of OsANN9-GFP were found in the plasma membrane, cytoplasm and nucleus (Figure 2B,C). This suggested that OsANN9 might be widespread in undifferentiated tissues.

Previous research results indicated that plant annexins could bind Ca^2+^ in participating physiological processes [24,26,27,28]. To investigate whether OsANN9 binds Ca^2+^, the plasmid pET30a-OsANN9-His was constructed and introduced into the *E. coli* BL21 strain. Meanwhile, the protein expression was induced by adding 0.5 mM isopropyl-β-D-thiogalactopyranoside (IPTG) at 18 °C. The OsANN9-His recombinant protein was evaluated by SDS-PAGE, with bands appearing at ~45KD that probably represent OsANN9 (Supplementary Figure S1A). To assay OsANN9 binding to Ca^2+^, the OsANN9-His recombinant protein was collected from the supernatant after lysing *E. coli* cells with ultrasonic waves. When different concentrations of Ca^2+^ (5 mM, 10 mM, 20 mM) were added to the supernatant, which contained an equal amount of OsANN9-His, precipitation occurred suddenly. After adding an equal amount of EDTA-Na_2_ (5 mM, 10 mM, 20 mM), precipitates redissolved due to the binding of EDTA-Na_2_ and Ca^2+^. SDS-PAGE demonstrated that for more OsANN9-His protein, a greater concentration of Ca^2+^ was added to the supernatant (Figure 2D). This result indicates that OsANN9 can bind Ca^2+^ in this manner.

To confirm that OsANN9 can bind Ca^2+^ in other scenarios, we performed the following tests. First, the OsANN9-His recombinant protein was purified with Ni-NTA affinity chromatography, and the eluted fraction was tested with a Western blot to ensure that OsANN9-His was represented by a band at ~45 KD (Supplementary Figure S1B,C). Second, to test the ability of OsANN9 to bind Ca^2+^, the fluorescence level of the OsANN9-His recombinant protein was assayed with a fluorescence spectrophotometer. The fluorescence intensity of purified OsANN9-His with or without Ca^2+^ was measured. The results showed that OsANN9-His reached a maximum fluorescence intensity of 5812 (A.U.) at 340 nm under excitation at 315 nm. After adding 2 mM Ca^2+^ to the OsANN9-His solution, the maximum fluorescence intensity increased to 6394 (A.U.) and decreased to 5526 (A.U.) at 340 nm after adding the same amount of EDTA-Na_2_ (Figure 2E). These results verified that OsANN9 can bind Ca^2+^ in this manner.

### 2.2. OsANN9 Expression Is Induced by Osmotic and Drought Stress

**Figure 3:**
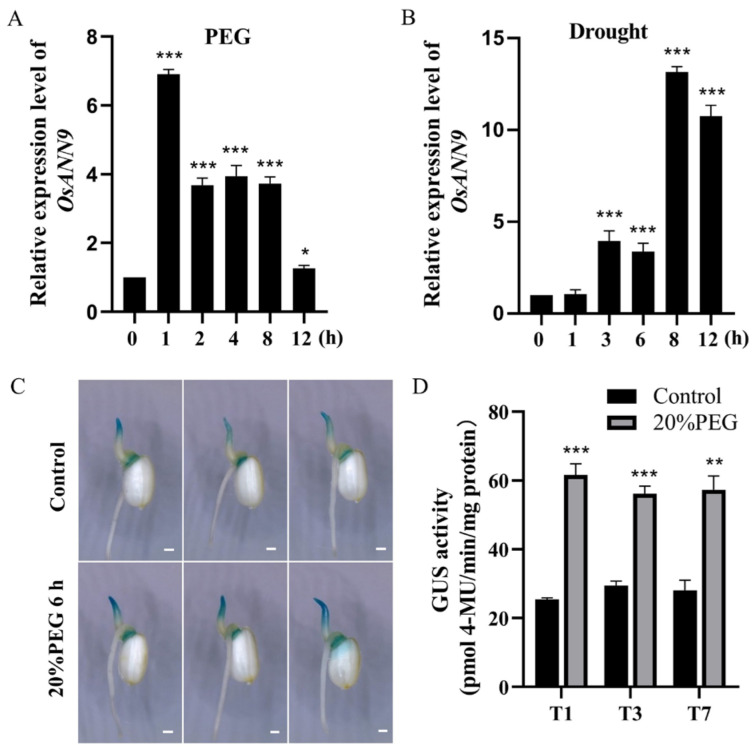
Expression pattern of *OsANN9* under PEG or drought treatment. (**A**,**B**) RT-qPCR results of *OsANN9* expression in WT seedlings under 20% PEG treatment and drought stress. *OsACTIN1* was used as the internal reference gene for normalization. (**C**) GUS staining of tissues from *OsANN9pro::GUS* transgenic rice lines. Bar = 1 mm. (**D**) Relative GUS activity with or without 20% PEG6000 for 6 h in *OsANN9pro::GUS* lines. T1, T3 and T7 were represented by three independent lines. Values are presented as the means ± SE of three biological replicates. * *p* < 0.05, ** *p* < 0.01, *** *p* < 0.001, Student’s *t*-test.

The previous studies indicated that OsANN1, OsANN3, OsANN4 and OsANN10 are involved in response to abiotic stresses [24,26,27,28]. It was hypothesized that OsANN9 may also have these kinds of properties. To explore the function of OsANN9, the 2277-bp promoter sequence upstream of *OsANN9* ATG was analyzed with the PlantCARE website (http://bioinformatics.psb.ugent.be/webtools/plantcare/html/) (accessed on 25 September 2020). The results showed that the *OsANN9* promoter sequence consists of a series of cis elements, including ABA-responsive elements (ABRE), v-myb avian myeloblastosis viral oncogene homolog (MYB), Dehydration Responsive Element (DRE) and MYB binding site (MBS), which might be controlled by abiotically induced transcription factors (Supplementary Figure S2). We further assessed the transcription level of *OsANN9* with PEG treatment in 14-day-old wild type (WT) plants. When treated with 20% PEG6000, the *OsANN9* transcript gradually increased approximately 7-fold compared to plants with no treatment (Figure 3A). These results suggested that *OsANN9* might have a key role in the response to PEG treatment. Moreover, we used the same WT seedlings to detect *OsANN9* transcription levels under drought stress. As expected, the results still showed that the *OsANN9* expression increased strikingly at 8 h; it amounted to a 13-fold increase compared to the no-drought treatment (Figure 3B).

To assess whether *OsANN9* was involved in the PEG treatment response, the plasmid *OsANN9pro::GUS* was introduced into *Agrobacterium tumefaciens* EHA105 and then transformed into rice calli. GUS staining and GUS activity were monitored in *OsANN9pro::GUS* plants under PEG-induced osmotic stress. The 3-day-old *OsANN9pro::GUS* rice plants were subjected to 20% PEG6000 treatment for 6 h, and GUS staining and GUS activity in seedlings were greatly increased following the 20% PEG6000 treatment (Figure 3C,D). Taken together, these results indicate that *OsANN9* positively responds to drought and osmotic stress in rice plants.

### 2.3. Construction of OsANN9-OE, OsANN9-Ri, and osann9 Mutants

**Figure 4:**
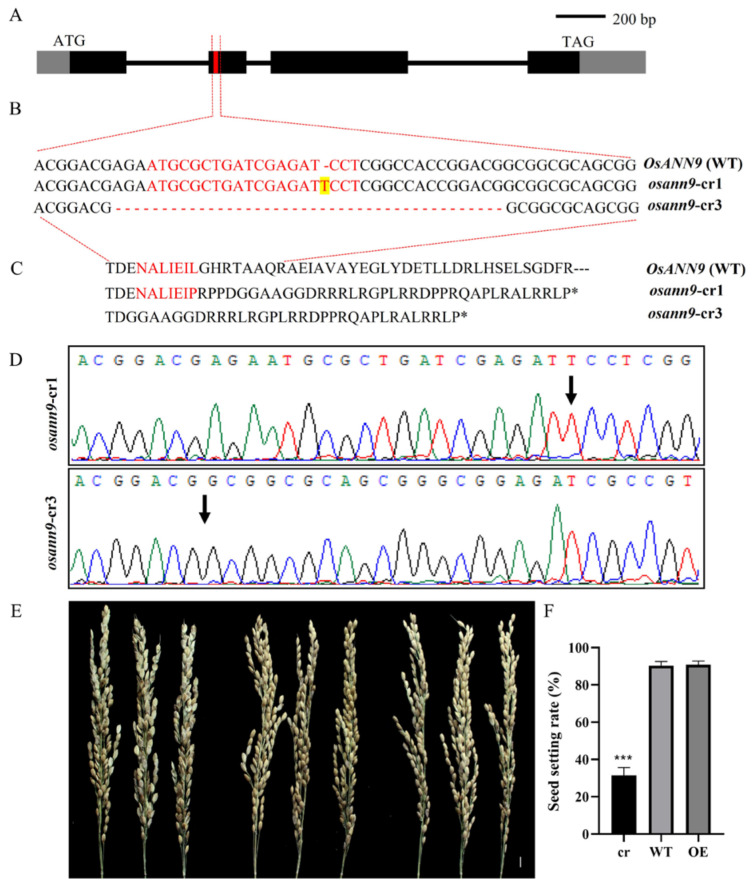
CRISPR-targeted mutagenesis of *OsANN9*. (**A**) Diagram of gene structure. Black boxes and lines indicate exons and introns, respectively, and gray and red boxes indicate UTRs and gRNA targeting regions, respectively. (**B**) The *osann9* mutants were generated by CRISPR/Cas9 gene editing. The sequence marked in red exhibits the gRNA target sequence. The yellow-labeled T shows the insertion site in the *osann9*-cr1 mutant. Short red lines show the deletion of 35-bp in the *osann9*-cr3 mutant. (**C**) Amino acid sequence alignment of OsANN9 in WT and osann9 mutants. The amino acids marked in red are encoded by the gRNA target sequence. Black asterisks represent premature protein termination. (**D**) DNA sequencing results showed an insertion of 1-bp T (the black arrow points) and a deletion of 35-bp (the black arrow points between two G) in the genomic DNA of *osann9*-cr1 and *osann9*-cr3. (**E**,**F**) Seed setting rate of *osann9*, WT and *OsANN9*-OE plant. Values are presented as the means ± SE of three biological replicates. n > 30. *** *p* < 0.001, Student’s *t*-test.

To explore the function of OsANN9 in response to osmotic stress, we isolated the 347-bp *OsANN9* CDS as the target region to construct the genetic vector for RNA interference (Supplementary Figure S3A). Twenty independent transgenic plants were obtained with a semblable phenotype. Meanwhile, the *Ubi::OsANN9-HA* expression cassette was constructed and inserted into plasmid pCAMBIA1300 (Supplementary Figure S3B), generating further rice *OsANN9* overexpression lines. The *osann9* mutant lines were generated by the *Agrobacterium*-mediated method. Two Cas9-free homozygous mutant lines (*osann9*-cr1 and *osann9*-cr3) were analyzed by DNA sequencing (Figure 4). In *osann9*-cr1, a 1-bp insertion was found at the 80 to 81st position upstream of the start codon, and another 1-bp insertion was found at the 596 to 597th position downstream of the start codon, resulting in a frameshift and a truncated protein with 123 aa. In *osann9*-cr3, a 35-bp deletion was found at the 578 to 612nd position downstream of the start codon, which led to a truncated protein with 111 aa (Figure 4C). After performing RT-qPCR, two RNAi lines (Ri2 and Ri7) with significantly reduced *OsANN9* transcript levels and two independent overexpression lines (OE2 and OE7) with prominently increased *OsANN9* transcript levels, as well as *osann9*-cr1 and *osann9*-cr3 mutant rice lines, were selected for subsequent studies (Supplementary Figure S4).

Very interestingly, the setting rates of *osann9* mutant rice plants significantly decreased in comparison to WT or *OsANN9*-OE plants (Figure 4E,F); the molecular mechanisms of this phenotype are still conducting. 

### 2.4. Overexpression of OsANN9 Activates the ROS Scavenging System, Thereby Enhancing Tolerance to Osmotic and Drought Stress

**Figure 5:**
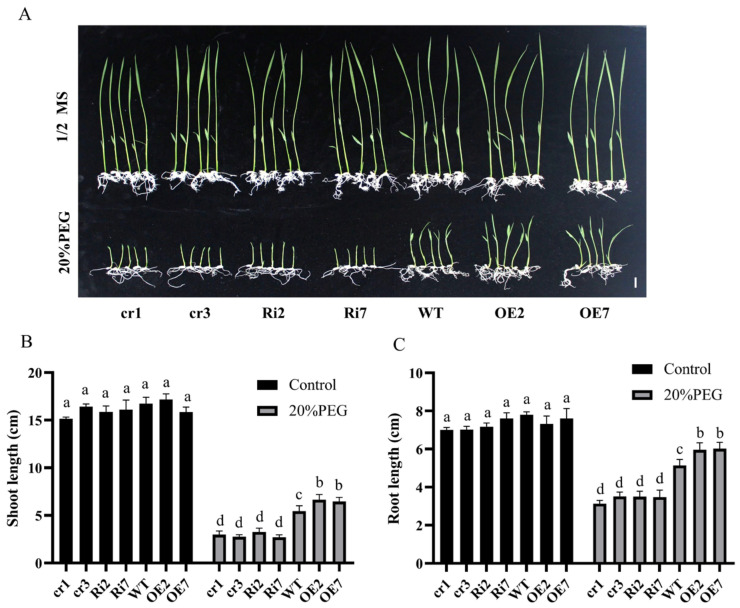
Osmotic stress test with *OsANN9*-OE, *OsANN9*-Ri, *osann9* and WT. (**A**) Performance of *OsANN9*-OE, *OsANN9*-Ri, *osann9* and WT seedings grown in 1/2 MS medium with or without 20% PEG6000 for 7 days. Bars = 1 cm. (**B**,**C**) Shoot and root length of plants in (**A**). Values are presented as the means ± SE of three biological replicates. n > 30. Different letters indicate significant differences among genotypes (on two-way ANOVA, *p* < 0.05).

To better understand the function of *OsANN9* in response to abiotic stress in rice, we applied osmotic stress to *OsANN9*-OE, *OsANN9*-Ri, *osann9* mutants and WT rice plants (Figure 5A). The above rice seeds were germinated on 1/2 Murashige and Skoog (MS) medium for 36 h. Then, the germinated seedlings were transferred to fresh 1/2 MS medium with or without 20% PEG6000. The lengths of shoots or roots were measured on the 7th day after treatment (Figure 5B,C). These measurements showed that the lengths of roots and shoots of *OsANN9*-OE seedlings on medium with 20% PEG6000 were strikingly longer than those of WT plants, while both *OsANN9*-Ri and *osann9* mutants showed weaker growth than WT plants. Meanwhile, no distinct differences were observed among different rice lines on the medium without 20% PEG6000. Overall, these observations suggest that *OsANN9* positively affects drought and osmotic stress responses in rice.

**Figure 6:**
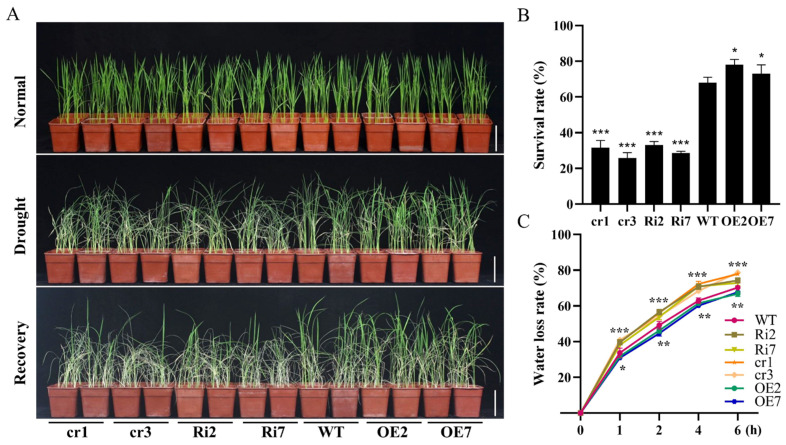
Drought-stress test with *OsANN9*-OE, *OsANN9*-Ri, *osann9* and WT. (**A**) Seedlings grown for 14 days under normal conditions (top panel) and were subjected to drought-stress for 10 days (middle panel), followed by re-watering for 7 days (bottom panel). Bars = 10 cm. (**B**) Survival rates of *OsANN9*-OE, *OsANN9*-Ri, *osann9* and WT plants after re-watering for 7 days. (**C**) Water loss by 14-day-old *OsANN9*-OE, *OsANN9*-Ri, *osann9* and WT whole plants. Fresh weight was monitored at the indicated times. Values are presented as the means ± SE of three biological replicates. * *p* < 0.05, ** *p* < 0.01, *** *p* < 0.001, Student’s *t*-test.

To further investigate the function of *OsANN9* in response to drought stress in rice, 14-day-old *OsANN9*-OE, *OsANN9*-Ri, *osann9* and WT rice seedlings were subjected to drought stress. The rice seedlings exhibited similar growth morphology under no stress conditions. After watering was stopped for 10 days, both *OsANN9*-Ri lines and *osann9* mutants showed hypersensitivity to drought and severe wilting, whereas the *OsANN9*-OE lines showed better growth comparing to the WT plants (Figure 6A). After 7 days of *osann9* recovery, 68% of WT plants survived. Meanwhile, the *OsANN9*-OE lines had the highest survival rate (78% for *OsANN9*-OE2 and 73% for *OsANN9*-OE7), and the *OsANN9*-Ri lines and *osann9* mutants exhibited the lowest survival rate (33% for *OsANN9*-Ri2, 28.6% for *OsANN9*-Ri7, 31.6% for *osann9*-cr1 and 25.8% for *osann9*-cr3) (Figure 6B).

In addition, to confirm the drought-stress response of OsANN9, the rate of water loss was assayed for the shoots of WT, *OsANN9*-OE and *OsANN9*-Ri lines as well as *osann9* mutant plants. Compared to WT rice plants, the rates of water loss of *OsANN9*-OE lines were lower at different time points; however, both *OsANN9*-Ri lines and *osann9* mutants had higher rates of water loss (Figure 6C). Based on the above results, the water loss of shoots matched the survival rate.

**Figure 7:**
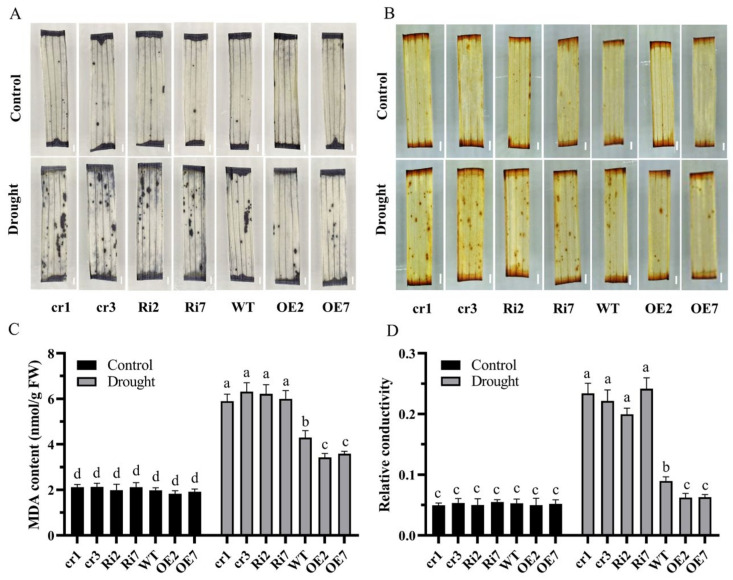
*OsANN9* overexpression reduced ROS accumulation and membrane lipid peroxidation. (**A**,**B**) In situ detection of H_2_O_2_ and O_2_^−^ in the leaves of transgenic plants under normal or drought-stress conditions with DAB and NBT staining. Bar = 1 mm. (**C**,**D**) MDA content and relative electrolyte leakage from the detached leaves of 14-day-old plants under normal or drought-stress conditions. Values are presented as the means ± SE of three biological replicates. n = 9; different letters indicate significant differences among genotypes (on two-way ANOVA, *p* < 0.05).

Drought stress may lead to the production of ROS [30], and high concentrations of ROS result in cell damage and even programmed cell death. As essential ROS, O_2_^.−^ and H_2_O_2_ have key roles in abiotic stress signaling. To confirm whether OsANN9 manipulates ROS levels in response to drought stress, nitro-blue tetrazolium (NBT) staining and 3,3′-diaminobenzidine (DAB) staining were performed on 7-day-old rice seedlings to assay the in situ localization of O_2_^−^ and H_2_O_2_, respectively. The results indicated that there were no differences in plants under normal conditions. However, when rice plants were drought-stressed for 4 h, blue spots (which reflect O_2_^−^ production) and brown spots (which reflect H_2_O_2_ production) were prominent in the mesophyll cells of *OsANN9*-Ri or *osann9* mutant plants, while fewer blue and brown spots were observed in WT and *OsANN9*-OE plants (Figure 7A,B). This result suggests that the production of ROS in rice might be related to the function of OsANN9 under drought.

**Figure 8:**
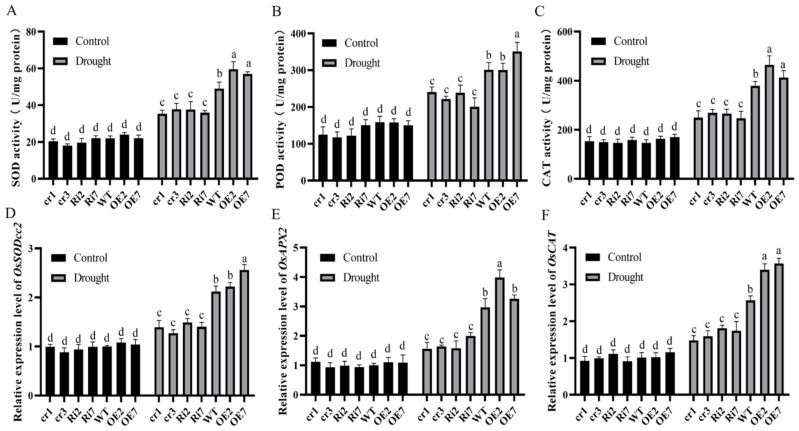
OsANN9 modulates ROS scavenging by regulating SOD, POD and CAT activity. (**A**–**C**) SOD, POD and CAT enzyme activity in 7-day-old plants under normal or drought-stress conditions. (**D**–**F**) The expression of *OsSODcc2*, *OsAPX2* and *OsCAT* in *OsANN9*-OE, *OsANN9*-Ri, *osann9* and WT plants under normal or drought-stress conditions. Values are presented as the means ± SE of three biological replicates. Different letters indicate significant differences among genotypes (on two-way ANOVA, *p* < 0.05).

The essential ROS scavengers SOD, POD and CAT eliminate excess cellular ROS, thus protecting plant cells from oxidative damage. To further clarify the role of *OsANN9* in regulating ROS under drought stress, SOD, POD and CAT activities were examined. The 7-day-old *OsANN9*-OE, *OsANN9*-Ri, *osann9* and WT rice lines were subjected to drought stress for 4 h. Interestingly, SOD, POD and CAT activities did not differ in any plants. After exposure to drought stress, SOD, POD and CAT were significantly increased in *OsANN9*-OE plants but decreased in *OsANN9*-Ri and *osann9* plants (Figure 8A–C). In addition, we also analyzed the transcription levels of ROS-scavenging genes, such as *OsSODcc2*, *OsAPX2* and *OsCAT*. The transcripts of these three genes were increased in the *OsANN9*-OE line and decreased in *OsANN9*-Ri and *osann9* plants under drought-stress conditions (Figure 8D–F). To sum up, both ROS scavenger activity and related gene expression levels were consistent with the drought tolerance phenotype of the *OsANN9* lines.

Both MDA content and electrolyte leakage are indicators of membrane damage and membrane lipid peroxidation [15,39]. According to the membrane lipid-binding ability of annexin, we examined MDA content and electrolyte leakage in *OsANN9*-OE, *OsANN9*-Ri, *osann9* mutant and WT plants under normal and drought-stress conditions. The MDA content in the above plants was similar under normal conditions; however, following drought treatment for 8 h, the average MDA content was 3.561 nmol/g/FW in *OsANN9*-OE plants, 6.108 nmol/g/FW in *OsANN9*-Ri plants and 6.218 nmol/g/FW in *osann9* mutants, while WT plants had an average MDA content of 4.299 nmol/g/FW. Furthermore, electrolyte leakage showed a similar pattern (Figure 7C,D).

## 3. Discussion

Drought stress is one of the most important environmental variables limiting plant growth and productivity [40], and generating drought-resilient crop varieties is essential for withstanding stress [41]. Annexins belong to a multifunctional protein family that exists in plants, animals and fungi [10]. Recent evidence has shown that plant annexins play critical roles in regulating abiotic and biotic stresses [19,21,25,38]. Our previous study showed that OsANN3 enhances tolerance to drought stress by modulating ABA-dependent stress response pathways in rice [26]. Bioinformatics analysis results indicated that OsANN9 consists of four annexin domains (Figure 1B); this suggests that OsANN9 may have functions in response to abiotic stress. Our results indicate that OsANN9 is involved in the process of ROS homeostasis during drought stress in rice. In addition, OsANN9 has a C2H2 domain, which implies that OsANN9 might have other properties which differ from other rice annexins.

In this study, we provide several lines of evidence that *OsANN9* is essential for drought tolerance in rice. Firstly, the expression of *OsANN9* was strongly induced by PEG treatment and drought stress, implying that *OsANN9* is a drought-response factor in rice (Figure 3A,B). Secondly, the *OsANN9*-OE rice plants demonstrated enhanced osmotic stress, as revealed by longer shoots and roots under PEG treatment, while the *OsANN9*-Ri and *osann9* mutant plants showed the opposite phenotype (Figure 5). Thirdly, *OsANN9* enhanced drought-stress tolerance in rice. The *OsANN9*-OE plants were much less sensitive to drought, as evidenced by lower levels of water loss, EL values, MDA content and ROS accumulation, and the *OsANN9*-OE plants had higher survival rates compared with WT plants under drought stress (Figure 6C and Figure 7). These results demonstrate that *OsANN9* is a promising candidate gene for developing drought-tolerance rice.

ROS production is usually triggered by and dramatically acclimates to drought stress [42]. Low levels of ROS can serve as a signal in regulating pathways for plant growth and stress responses. Excessive ROS accumulation causes oxidative damage to proteins and damage to lipids in the cell membrane [43,44]. In this study, overexpression of *OsANN9* reduced the accumulation of ROS caused by drought stress, as illustrated by lower DAB and NBT staining levels in *OsANN9*-OE plants. This result suggested that O_2_^−^ and H_2_O_2_ contents in *OsANN9*-OE plants were lower than those in WT plants under drought stress (Figure 7A,B). Antioxidant protection is an effective way to shield plants from stress-induced oxidative damage. In this study, SOD, POD and CAT activities under drought stress were markedly increased in *OsANN9*-OE plants compared to *OsANN9*-Ri and *osann9* mutant plants (Figure 8A–C). Therefore, OsANN9 may improve drought-stress tolerance by modulating the intracellular level of H_2_O_2_.

In addition, some stress biomarkers have been widely used in detecting and monitoring stress tolerances in plant, such as water content, water-use efficiency, hydraulic conductivity, photosystem II (PSII) and photosystem I (PSI) efficiency, and so on [45]. For example, as an essential photosynthetic parameter, electron transport rate (ETR) affects crucial traits such as stress tolerance, especially drought tolerance [46,47].

Calcium ion (Ca^2+^) is a ubiquitous intracellular second messenger that mediates plant responses to abiotic stress [48]. Annexins are a family of calcium-dependent membrane-binding proteins that can sense and regulate [Ca^2+^]_cyt_ under abiotic stress [28,49,50]. It has been reported that AtANN4 can be phosphorylated by AtSOS2, mediating salt-induced increases [Ca^2+^]_cyt_ in *Arabidopsis* [51]. *OsANN4* overexpression can lead to increasing Ca^2+^ influx in response to ABA in rice [27]. Based on bioinformatics predictions, OsANN9 has two type-II Ca^2+^-binding sites (Figure 1B). The results of in vitro assays also showed that OsANN9 has Ca^2+^-dependent phospholipid-binding activity and a Ca^2+^-binding capacity (Figure 2D,E). Plant hormones are small-molecule compounds widely present in plants. Many endogenous hormones such as ABA, gibberellins (GA), and cytokinin (CYT) are involved in regulating plant growth and development and play crucial rules in stress resistance [52]. For instance, drought stress could suppress GA biosynthesis, which leads to SLR1 accumulation and thus inhibits the degradation of OsPYL10, finally enhancing the ABA signal response to drought stress [53]. Interestingly, SnRK2s were activated by ABA and then phosphorylated the type-A *Arabidopsis* response regulator (ARR5) proteins, which strengthen the ABA signal under drought stress; however, the cytokinin signal could be repressed by type-A ARR5, which restricts plant development [54]. Nevertheless, the exact mechanism underlying how OsANN9 modulates Ca^2+^ and plant hormones under drought stress requires further exploration. 

Based on our results, drought stress increased the expression of *OsANN9*, thus enhancing the drought tolerance of rice. OsANN9 not only heightened ROS scavenger activity but also alleviated cell damage upon drought stress. In conclusion, we postulate that *OsANN9* is a candidate gene for breeding rice cultivars with tolerance to drought stress. However, we cannot yet specify the exact mechanism by which the OsANN9 protein contributes to drought-stress responses via Ca^2+^ signaling. Future studies must identify other signaling pathways to fully understand the role of OsANN9 in plant physiological processes. In a word, we first constructed and used CRISPR lines of rice *OsANN9* and further demonstrated that OsANN9 might be involved in a few mechanisms for drought-stress responses.

## 4. Materials and Methods

### 4.1. Plant Materials and Growth Conditions

The rice (*Oryza sativa* subsp. *japonica*) cultivar *Nipponbare,* which was planted in the experimental field at Hebei Normal University (Shijiazhuang, China, 38°02′33″ N 114°30′36″ E), was used in this research. Rice plants were grown on 1/2 MS solid medium in a chamber or greenhouse at temperatures of 28/22 °C (day/night) with 60–70% humidity under 14/10 h light/dark cycles.

To perform the PEG treatment assay, rice seeds were planted on 1/2 MS solid medium with or without 20% (*w*/*v*) PEG6000 for 7 days. Then, a photograph was taken, and the length of the shoots or roots was measured.

For drought tolerance analysis, rice seeds were sown on 1/2 MS solid medium and grown for 7 days before being transferred to soil and grown in pots (12 × 12 cm) for 7 days with regular water. Then, all plants were dried for 10 days and given a 7-day recovery.

To assay water loss, 14-day-old rice plants were placed on filter paper at room temperature. The weight of the plants was measured at different time points. The water loss rate was calculated as the ratio of total weight lost compared to the initial weight at different time points [55].

### 4.2. Construction of OsANN9 Expression Vectors

To overexpress *OsANN9*, the CDS of *OsANN9* was inserted into the pCAMBIA1301-Ubi::HA expression vector, using the *Bam*HI site. To construct an RNA interference construct, a 347-bp cDNA fragment of *OsANN9* was cloned into the pTCK303 vector using the *Kpn*I and *Bam*HI sites. To construct the CRISPR/Cas9 vector, the specific sgRNA sequences of *OsANN9* were synthesized, and the CRISPR/Cas9 vector was generated using the genome editing approach, as reported previously [56].

To construct the *35S::OsANN9-GFP* vector, the CDS of *OsANN9* was cloned into the pMDC83 vector using the *Xba*I and *Bam*HI sites. For the *OsANN9pro::GUS* vectors, the 1879-bp genomic sequence upstream of the *OsANN9* ATG was cloned into the pCAMBIA1300-GUS vector using the *Xba*I and *Pst*I sites.

All constructs were introduced into *Agrobacterium* strain EHA105 separately and subsequently transformed into *Nipponbare* by the *Agrobacterium*-mediated method [57]. All primers for vector construction are listed in Supplementary Table S1.

### 4.3. Subcellular Localization of OsANN9

Root tips of 3-day-old OsANN9-GFP rice seedlings were used to observe subcellular localization with a fluorescence confocal microscope. *Agrobacterium* GV3101 carrying the *35S::OsANN9-GFP* or *35S::GFP* plasmid was injected into tobacco leaves. After 40 h of infiltration, fluorescence signals were observed using a fluorescence confocal microscope. The *35S::OsANN9-GFP* or *35S::GFP* construct was transformed into rice protoplasts via the PEG-mediated method [58], and the fluorescence signals were observed with a fluorescence confocal microscope (FV3000, Olympus, Tokyo, Japan).

### 4.4. Recombinant OsANN9-His Protein Purification and Ca^2+^-Binding Activity

The CDS of *OsANN9* was inserted into a pET30a vector to generate OsANN9-His constructs. The construct was transformed into the *E. coli* strain BL21, and recombinant protein OsANN9-His was induced by adding 0.5 mM IPTG at 18 °C for 6 h. The *E. coli* cells containing OsANN9-His were lysed via ultrasonication, and samples were ultracentrifuged at 12,000× *g* for 10 min at 4 °C. The supernatant was used for the Ca^2+^-binding activity assay, as described previously [24]. Different concentrations of CaCl_2_ (5 mM, 10 mM, 20 mM) and an equivalent amount of EDTA-Na_2_ were added successively to the supernatant. SDS-PAGE was used to assay the protein samples.

For fluorescence measurements, the recombinant protein OsANN9-His was purified using Ni-NTA affinity chromatography. The assay medium contained 0.2 mg mL^−1^ OsANN9-His protein and 0 mM or 2 mM Ca^2+^ in the buffer (20 mMTris-HCl, pH 8.0). Fluorescence spectroscopy was performed using a fluorescence spectrophotometer (F-4600, Hitachi, Tokyo, Japan).

### 4.5. GUS Staining and GUS Activity

*OsANN9pro::GUS* seedlings were incubated with 5-bromo-4-chloro-3-indolyl-β-glucuronic acid buffer at 37 °C under dark conditions for GUS staining. Then, the stained seedlings were immersed in 70% ethanol to remove surface dyes and chlorophyll. For GUS activity detection, the protein was extracted from *OsANN9pro::GUS* seedlings and incubated with 4-methylumbelliferyl β-D-glucuronide (MUG). The cleavage of MUG was monitored quantitatively.

### 4.6. Antioxidant Enzyme Activity

The 7-day-old rice seedlings were homogenized in phosphate buffer (50 mM phosphate, 1 mM EDTA-Na_2_, 1% (*w*/*v*) polyvinyl pyrrolidone, pH 7.4) and centrifuged for 30 min at 4 °C at 10,000× *g*. The supernatant was used for the antioxidant enzyme activity assay. The CAT, POD and SOD activities were tested as reported previously [59].

### 4.7. Detection of O_2_^−^ and H_2_O_2_ In Situ

To detect O_2_^−^ in situ, the leaves of 7-day-old rice seedlings were detached and immersed in 6 mM NBT for 24 h at 25 °C. Then, leaves were boiled in ethanol for 30 min to remove chlorophyll. The samples were imaged with a 3D microscope (DVM6, Leica, Wetzlar, Germany).

For the detection of H_2_O_2_ in situ, DAB staining was performed on the leaves of 7-day-old rice seedlings. The leaves were immersed in DAB solution for 12 h at 25 °C. To remove chlorophyll, the leaves were incubated with ethanol. Samples were imaged with a 3D microscope (DVM6, Leica, Wetzlar, Germany).

### 4.8. Measurement of MDA and Electrolyte Leakage

The 14-day-old rice seedlings were homogenized in 0.1% (*w*/*v*) trichloroacetic acid (TCA) and then centrifuged at 1500 g for 10 min at 4 °C. The MDA content was determined with thiobarbituric, as reported previously [60].

For the electrolyte leakage assay, 7-day-old rice seedlings were soaked in deionized water for 24 h, and this conductivity was measured as E1. Then, the samples were boiled for 30 min, and conductivity after cooling was measured as E2. The conductivity of deionized water was measured as E0. This conductivity measurement method was described previously [28].

## Data Availability

The data presented in this study are available on request from the corresponding author.

## Supplementary Materials

The following supporting information can be downloaded at: https://www.mdpi.com/article/10.3390/ijms242417495/s1.
