# Supplementary material for: Rice OsANN9 Enhances Drought Tolerance through Modulating ROS Scavenging Systems
Source: Int J Mol Sci. 2023 Dec 15;24(24):17495. doi: 10.3390/ijms242417495 (PMC10743917; doi:10.3390/ijms242417495)
Supplement: Supplementary file 1 [file ijms-24-17495-s001.zip › ijms-2745881-supplementary.pdf]

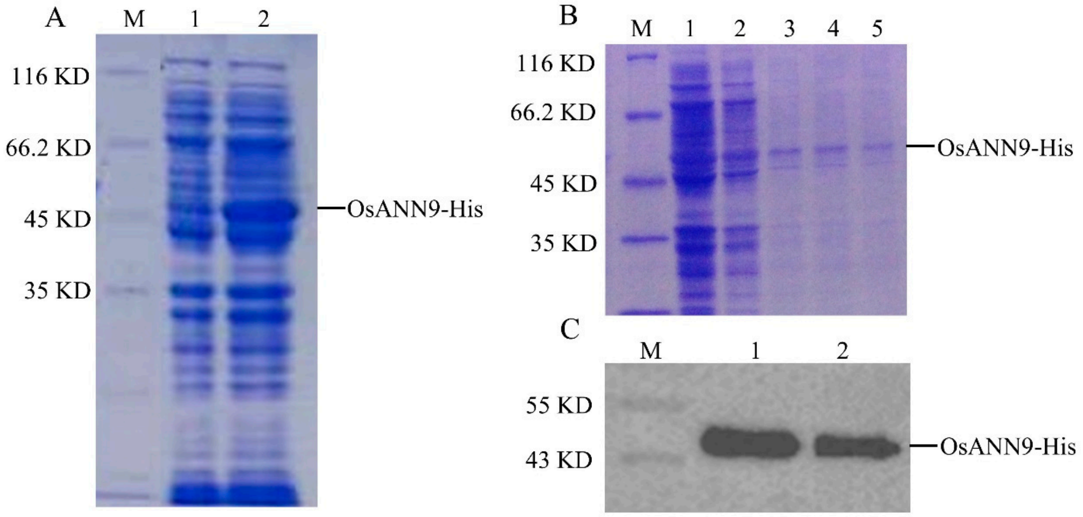

Figure S1 Purification of OsANN9-His protein. (**A** and **B**) Induction and purification of OsANN9-His protein. pET30a-OsANN9 was introduced into *E. coli* BL21 strain (lane 1 in A) and induced with 0.5 mM IPTG at 18°C for 6 h (lane 2 in A). OsANN9-His protein purification was performed with 0.5 mL elution buffer containing 250 mM imidazole (lane 2 in B) and 500 mM imidazole (lane 3-5 in B). (**C**) Western blot analysis of OsANN9-His protein. M: protein molecular weight marker.

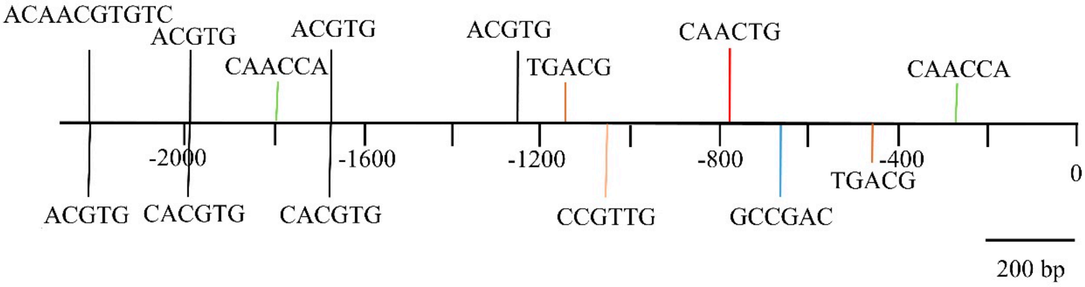

Figure S2 Prediction of stress-related cis-acting elements in the promoter sequence of *OsANN9*. Six CRE types, including ABRE (black line), DRE (blue line), MBS (red line), MYB-recognition site (yellow line), MYB (green line) and TGACG-motif (brown line) were universally distributed in the *OsANN9* promoter regions and are shown with different colored lines.

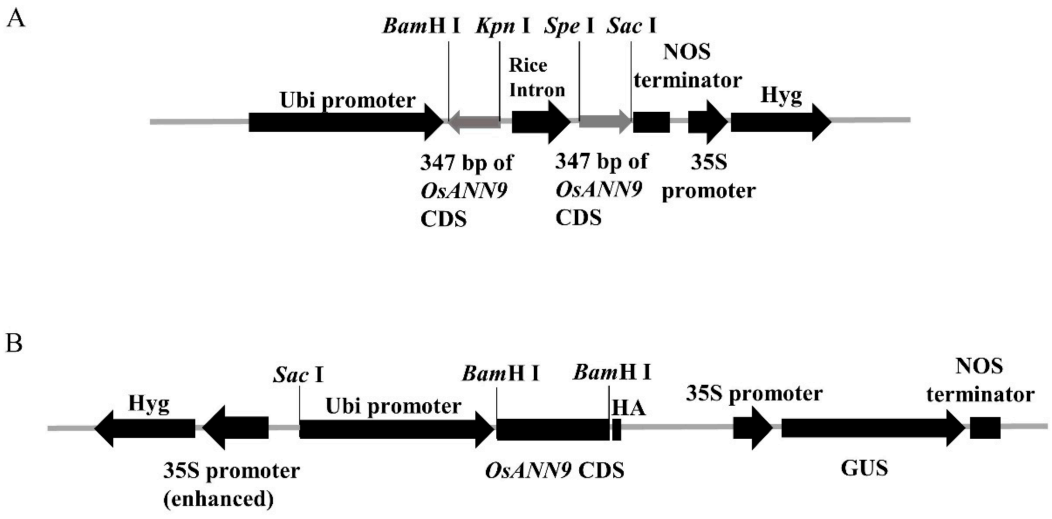

Figure S3 Schematic diagram of expression vectors. (**A**) Schematic diagram of the specific CDS fragment of *OsANN9* in the pTCK303-*OsANN9*-RNAi vector. (**B**) Schematic diagram of the CDS of *OsANN9* in the pCAMBIA1301-Ubi::HA expression vector.

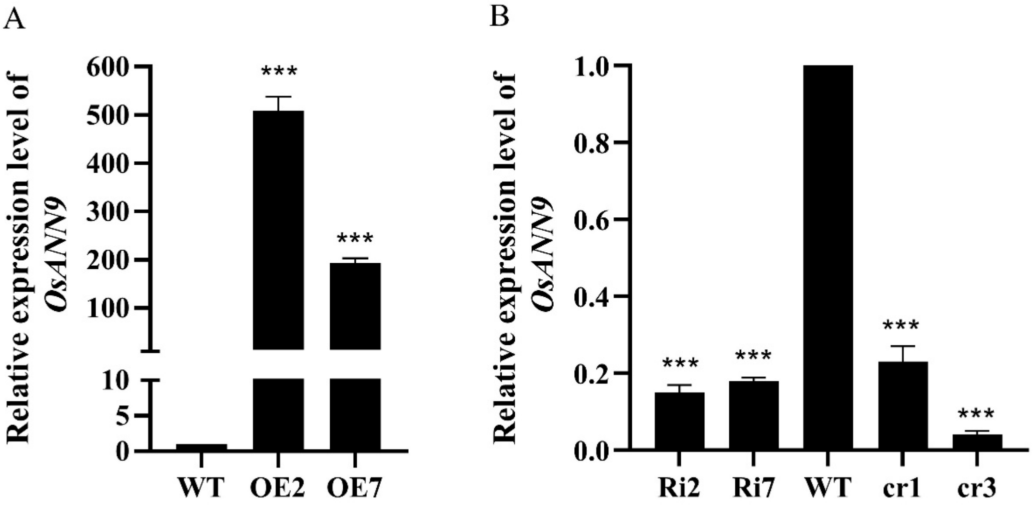

Figure S4 Transcript levels of *OsANN9* in WT, *OsANN9*-OE, *OsANN9*-Ri and *osann9* plants. (**A** and **B**). The RT-qPCR results of *OsANN9* expression level in the WT, *OsANN9*-OE (OE2, OE7), *OsANN9*-Ri (Ri2, Ri7) and *osann9* (cr1, cr3) plants. The expression level in WT was set to 1. Values were means ± SD of three replicates. *P < 0.05, **P < 0.01, ***P < 0.001. Student's *t*-test.

Table S1 Primer sequences used in plasmids construction and PCR

Primers	Sequence	Purpose
P1	5' TTCTGCAGCGGGATCCATGTGTTGCTGGTGCTGCTG 3'	*OsANN9* cDNA
P2	5' CGTAACGCGTGGATCCTTGGTACCCTTCTCAGGGCCGACGA 3'	amplification
P3	5' GGTACCACTAGTGGGTAGAGCCCAGAGCATTG 3'	*OsANN9* RNAi
P4	5' GGATCCGAGCTCGATCAGCGCATTCTCGTCC 3'	fragment amplification

P5	5' GCCGAGGGTAGAGCCCAGAGCAT 3'	CRISPR-sgRNA1-
P6	5' AAACATGCTCTGGGCTCTACCCT 3'	*OsANN9*
P7	5' GTTGATGCGCTGATCGAGATCCT 3'	CRISPR-sgRNA2-
P8	5' AAACAGGATCTCGATCAGCGCAT 3'	*OsANN9*
P9	5' CTGCAGAACTCAGATGGGCTACAGAA 3'	*OsANN9* promoter
P10	5' ACTAGTGGCACACACACGCCTCCTACGC 3'	amplification
P11	5' CATCTCTCAGCACATTCCAGCAG 3'	RT-qPCR
P12	5' AGGAGGACGGCGATAACAGC 3'	(*ACTIN1*)
P13	5' CTCCATTTCTCCCCCCATTCTCT 3'	RT-qPCR
P14	5' TTCTCGTCCGTTCCCCATCCTT 3'	(*OsANN9*)
P15	5' TAAGGGCACTATCCACTTTGTCC 3'	RT-qPCR
P16	5' ACCAGCATGGCGGGTCTCA 3'	(*OsSODcc2*)
P17	5' ACCGCCTCGGACCAAACTAC 3'	RT-qPCR
P18	5' GGCAATCACCACCTTCTCGC 3'	(*OsCAT*)
P19	5' CCTTCTTCAGCTGCCAAGTG 3'	RT-qPCR
P20	5' ATCGGCATTAATCTTTIGCGG 3'	(*OsAPX2*)
